# Selenium Nanoparticles Decorated by Blueberry Pomace Polysaccharides Improve the Protection Effects Against Erythrocyte Hemolysis

**DOI:** 10.3390/foods15020299

**Published:** 2026-01-14

**Authors:** Ling Zhu, Yinzhao Gao, Yaqin Xu, Conglei Ma, Xindi Zhang, Yaxi Han, Libo Wang, Lijun Guan

**Affiliations:** 1 College of Arts and Sciences, Northeast Agricultural University, Harbin 150030, China; zhuling1998@outlook.com (L.Z.); s231102002@neau.edu.cn (Y.G.); xuyaqin@neau.edu.cn (Y.X.); s241101021@neau.edu.cn (C.M.); 2 Heilongjiang Province Academy of Agricultural Sciences Institute of Food Processing, Harbin 150086, China; zhangxindi97@163.com (X.Z.); navycki@163.com (Y.H.)

**Keywords:** blueberry pomace, polysaccharides, selenium nanoparticles, structural characterization, hemolysis

## Abstract

In this study, selenium nanoparticles (SeNPs) were synthesized using polysaccharides extracted from blueberry pomace (BP) as a stabilizing agent. BP was characterized as an acidic polysaccharide with a molecular weight of 5.4 × 10^5^ Da. The resulting BP-SeNPs were monodisperse spheres with an average size of 94.33 nm, as confirmed by TEM, DLS, FT-IR, XRD, and EDX analyses. Compared to SeNPs, BP-SeNPs demonstrated superior stability under varying conditions of storage time, temperature, pH, and ionic strength. Furthermore, in vitro evaluation using AAPH-induced rabbit erythrocytes revealed that BP-SeNPs offered enhanced protection against hemolysis. This protective effect was attributed to their ability to significantly bolster antioxidant enzyme activities (SOD, CAT, and GSH-Px) and preserve membrane integrity by maintaining ATPase function and sialic acid content. These results establish BP as an effective stabilizer for SeNPs and suggest the promising potential of BP-SeNPs as antioxidant agents in functional food or nutraceutical applications.

## 1. Introduction

Nanoparticles are typically defined as particles with a length of 1–100 nanometres [1]. They can be manufactured from a variety of materials, including metals, semiconductors, polymers, and ceramics, giving rise to distinct classes such as metallic nanoparticles (e.g., gold, silver), carbon-based nanomaterials (e.g., carbon dots, graphene oxide), polymeric nanoparticles, and lipid-based nanocarriers [2,3,4]. The large surface-to-volume ratio and high surface energy of nanoparticles confer broad application prospects in the food and pharmaceutical fields [5]. Selenium (Se) is an essential trace element for the human body, playing a vital role through its various physiological and pharmacological functions, such as antioxidant activity, anticancer, and immunity enhancement [6]. To ensure adequate selenium intake, organic selenium compounds, such as selenomethionine and selenocysteine, and inorganic selenium compounds, such as selenate and selenite, are widely used as supplements [7]. Nevertheless, the Se dosage is severely restricted on account of its toxic effect. As a new form of Se, the red zero-valent selenium nanoparticles (SeNPs) have attracted much attention because of their higher bioactivity, bioavailability, and much lower toxicity than inorganic selenium compounds [8]. Compared to other nanomaterials that suffer from limitations such as non-biodegradability (carbon-based nanoparticles), costly production processes, and complex stabilization procedures (liposomal nanoparticles and polymeric nanoparticles), SeNPs demonstrate greater competitiveness due to their excellent biodegradability, safety profile, and environmentally friendly synthesis pathways [9]. However, SeNPs are unstable and prone to aggregate due to high surface energy. To enhance nanoparticle stability during preparation, storage, and application, stabilizers such as polyphenols, polysaccharides, and proteins are commonly employed [10]. Many studies have reported that SeNPs covered with polysaccharide molecules showed better activities than pure SeNPs. For instance, Nie et al. [11] found that *Catathelasma ventricosum* polysaccharides SeNPs had higher antidiabetic activity than other selenium preparations including SeNPs, selenocysteine, and sodium selenite. Yu et al. [12] confirmed that a nano-selenium derivative of *Stauntonia brachyanthera* pulp polysaccharide significantly inhibited the proliferation of HepG2 cells and triggered apoptosis in HepG2 cells through the mitochondrial pathway. Therefore, SeNPs stabilized by polysaccharides had excellent potential in functional foods and disease prevention.

Blueberry (*Vaccinium* spp.), belonging to the Ericaceae family, has been recognized as a precious “superfood” for its unique flavour and diverse health-promoting nutrients, and its fruits are rich in bioactive compounds, such as phenolic compounds, polysaccharides, vitamin, and organic acids [13]. In the past five years, blueberry fruit polysaccharides have received extensive attention for their biological activities. For example, Wang et al. [14] prepared three polysaccharides from blueberries with hypolipidemic and immunological activities. Qiao et al. [15] extracted three pectin polysaccharides from blueberries with great antioxidant and antibacterial activities and found that better bioactivities were significantly correlated with lower molecular weight. It was reported that the blueberry pomace after fresh fruit processing still contained abundant water-soluble polysaccharides. Arland et al. [16] found that the blueberry pomace contained 46.2% polysaccharides and 21.59% reducing sugars after processing. Based on these reports, the possible applications of blueberry pomace polysaccharides should be further studied and utilized. The synthesis of SeNPs using polysaccharides as stabilizing agents is conducted under mild conditions in an aqueous medium without toxic chemicals, aligning with green synthesis principles and representing an environmentally friendly preparation strategy [17]. In addition, plant polysaccharides possess various biological activities, including antitumor, antioxidant, antidiabetic, hypolipidemic, and antimicrobial effects [18]. Currently, blueberry extracts have been successfully utilized in the green synthesis of metal nanoparticles (gold, silver, copper, etc.) due to their strong reducing capacity, highlighting the potential of blueberry resources in the field of nanotechnology [19,20]. As far as we know, there has been no study about the usage of the blueberry pomace polysaccharides as a dispersing agent for SeNPs preparation. Therefore, this research direction should be considered as a priority.

Erythrocytes are especially prone to oxidative stress owing to their high content of polyunsaturated fatty acids in the membrane and auto-oxidation of hemoglobin [21]. Consequently, exposure of erythrocytes to free radicals induces lipid peroxidation, protein cross-linking, and thiol oxidation, ultimately leading to cell membrane damage and hemolysis [22]. Nanotechnology-based antioxidants, especially SeNPs, offer great promise due to their potent antioxidant properties and low toxicity. When stabilized by plant-derived polysaccharides, SeNPs gain improved stability and synergistic bioactivity, highlighting their potential as a natural and effective strategy to protect erythrocytes from oxidative damage [23]. The objective of this study was to prepare SeNPs stabilized by the polysaccharide from blueberry pomace (BP) and determine the protective effect of SeNPs as an antioxidant against erythrocyte hemolysis. The structural and morphological properties of BP-SeNPs were systematically characterized; the stabilities of BP-SeNPs under different conditions were determined. In addition, the mechanism of BP-SeNPs against hemolysis was evaluated in vitro using rabbit erythrocytes.

## 2. Materials and Methods

### 2.1. Materials and Reagents

Blueberry pomace was purchased from Luye Berry Co. (Harbin, China), ground into a powder after dehydration, packed in an airtight polythene bag, and stored at −18 °C. D101 macroporous resin and standard dextrans were acquired from Baierdi Biotechnology Co. (Beijing, China). Ascorbic acid (VC) and sodium selenite were provided by Zhiyuan Chemical Reagent Co., Ltd. (Tianjin, China). Standard monosaccharides were bought from Sigma Aldrich, Inc. (Saint Louis, MO, USA). Rabbit erythrocyte (2%) was purchased from Hongquan Biotechnology Co. (Guangzhou, China). 2,2′-Azobis (2-methylpropionamidine) dihydrochloride (AAPH), trifluoroacetic acid (TFA), and 1-phenyl-3-methyl-5-pyrazolone (PMP) were purchased from Aladdin Reagent Co. (Shanghai, China). Assay kits for determination of malondialdehyde (MDA), sialic acid (SA), catalase (CAT), superoxide dismutase (SOD), glutathione peroxidase (GSH-Px), and ATPase (Na^+^/K^+^-ATPase, Ca^2+^-ATPase, and Mg^2+^-ATPase) were purchased from Abcam (Cambridge, UK). Other reagents were of analytical grade.

### 2.2. Extraction and Purification of Blueberry Pomace Polysaccharides

Ultrasonic-assisted ammonium sulfate/ethanol aqueous two-phase system was used to extract the crude polysaccharides from blueberry pomace, according to our previous report [24]. Ammonium sulfate (14.0 g) was added to 30.0 mL of deionized water; then, 20.0 mL of ethanol was mixed. Blueberry pomace was added into the above system with the material-to-liquid ratio of 1:80 g/mL and treated in an ultrasonic bath (JY92-2D, Ningbo Scientz Biotechnology Co., Ltd., Ningbo, China) at 340 W for 39 min. After extraction, the bottom phase solution was dialyzed (*M*_w_ cut-off 3500 Da) at room temperature for 48 h to remove salt. The dialysate was collected, concentrated to one-fifth of its original volume, and mixed with four volumes of 75% ethanol. The mixture was then allowed to precipitate at 4 °C overnight. The precipitate was redissolved in deionized water and lyophilized to obtain the crude polysaccharide. D101 macroporous resin column (2.5 × 60 cm) was used to purify the crude polysaccharides (20 mL, 2 mg/mL) with deionized water as eluant at a flow rate of 1.0 mL/min. The total elution volume used was 800 mL. All the eluates obtained were collected, concentrated, and freeze-dried to gain blueberry pomace polysaccharide, labelled as BP.

### 2.3. Determination of Physicochemical Properties and Structure of BP

#### 2.3.1. Chemical Components

Total sugar content was measured by the phenol–sulphuric acid method using d-glucose as a standard [25]. Reducing sugar content was investigated according to the dinitrosalicylic acid method [26]. Uronic acid content was assessed using carbazole–sulfuric acid method [27]. Protein content was estimated by the Bradford method [28]. Polyphenol content was determined by the Folin phenol method [29]. The purity of the polysaccharide BP was calculated as the total sugar content minus the reducing sugar content.

#### 2.3.2. Molecular Weight

Based on the method previously reported by Zhao et al. with slight modification [30], the molecular weight (*M*_w_) of BP was tested by high-performance liquid chromatography (HPLC, Waters 2695/2414, Waters Co., Milford, MA, USA) furnished with Waters Ultrahydrogel Linear column (7.8 × 300 mm) and differential refraction index detector (Waters-2414, Waters Co., Milford, MA, USA). Ultrapure water was applied as mobile phase at a flow rate of 0.6 mL/min. The polysaccharide solution (10 μL, 1 mg/mL) was filtered through a membrane (0.22 μm, MilliporeSigma, Burlington, MA, USA) and then tested. The molecular weight of the polysaccharide was calculated using the calibration curve made by dextran standards (5, 10, 40, 70, and 110 kDa).

#### 2.3.3. Monosaccharide Composition

The monosaccharide composition of BP was carried out according to the previous study [31]. BP (10.0 mg) was put into a sealed ampoule bottle and hydrolyzed with 6.0 mL TFA (2 mol/L), using high temperature over 6 h at 110 °C. After cooling to room temperature, 4 mL of methanol was added, and the solution was evaporated to dryness in a rotary evaporator (50 °C). This process was repeated three times to remove excess TFA. Afterward, the hydrolysate was mixed with 0.9 mL of NaOH (0.3 mol/L) and 0.9 mL of PMP methanol solution (0.3 mol/L). After vortex mixing for 2 min, the mixture was incubated in a water bath at 70 °C for 90 min, cooled to room temperature, and neutralized by adding 0.9 mL of HCl (0.3 mol/L). Finally, 5.0 mL of chloroform was added to the resulting mixture and vortexed for 2 min. After standing, the chloroform phase was discarded to remove PMP. Subsequently, the sample was filtered through a 0.22 μm organic microporous filter membrane. A series of monosaccharide standards (mannose, rhamnose, glucuronic acid, galacturonic acid, glucose, galactose, and arabinose) were derivatized following the same procedure. The concentration of each standard solution was 2 mg/mL. The sample and monosaccharide standards were determined by HPLC (Waters 2695/2414, Waters Co., Milford, MA, USA) combined with a C18 column (4.6 × 150 mm) and an ultraviolet absorption detector (Waters 2487/2489, Waters Co., Milford, MA, USA). The injection volume was 10 μL, and elution was performed for 30 min using 0.01 mol/L of phosphate buffer (A) and acetonitrile (B) (A:B = 83:17, *v/v*) as the mobile phase, at a flow rate of 0.6 mL/min.

#### 2.3.4. FT-IR Spectroscopy

BP (2 mg) was mixed with 100 mg KBr powder and pressed into a disc. The sample was recorded using FTS135 FT-IR spectrometer (Bio-Rad Laboratories Co., Hercules, CA, USA) in the frequency range of 4000–400 cm^−1^.

#### 2.3.5. NMR Spectra

BP (40 mg) was dissolved in 1.0 mL of D_2_O under ultrasound assistance and then centrifuged at 17,888× *g* for 10 min (4 °C). Then, 0.5 mL of supernatant was recorded on an AVANCE III 600 MHz spectrometer (Bruker Corporation, Billerica, MA, USA) at room temperature, including ^1^H-NMR, ^13^C-NMR, heteronuclear single quantum coherence (HSQC), correlation spectroscopy (COSY), and heteronuclear multiple bond correlation (HMBC).

### 2.4. Preparation of BP-SeNPs

**Figure 1:**
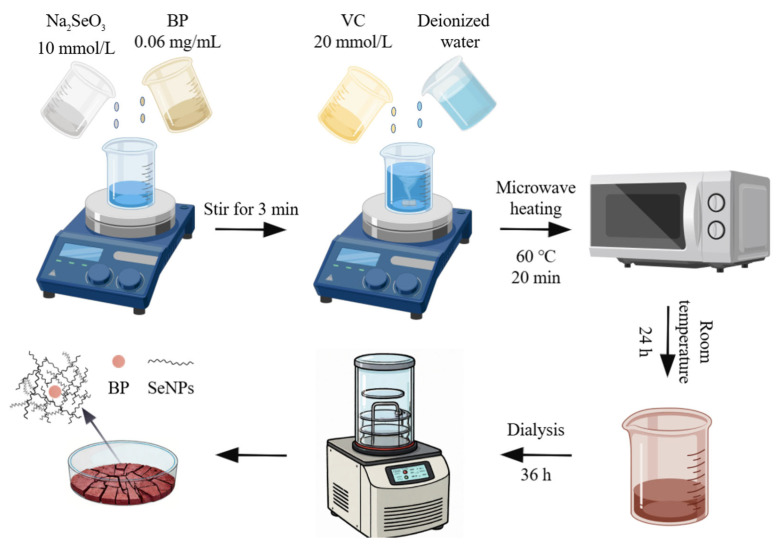
Synthetic process flowchart.

The SeNPs stabilized by BP were synthesized according to the method of Yang et al. with some modifications [32]. The Na_2_SeO_3_ (10 mmol/L, 2.0 mL) solution was slowly added to the BP solution (0.06 mg/mL, pH 7.4), and the mixture was gently stirred for 3 min. After that, 2.0 mL of 20 mmol/L VC was added into the mixture and diluted to 20.0 mL with deionized water. The mixture was reacted in a microwave (XH-800C, Xianghu Technology Development Co., Ltd., Beijing, China) at 60 °C (100 W) for 20 min and placed at room temperature for 24 h. Then, the resulting solution was dialyzed with deionized water (*M*_w_ cut-off 3500 Da) for 36 h, concentrated, and lyophilized to obtain selenium nanoparticles (BP-SeNPs). The synthetic process flowchart is shown in Figure 1.

As a negative control, pure selenium nanoparticles without BP were prepared under the same conditions and named SeNPs. The average particle size, polydispersity index (PDI), and zeta potential of SeNPs and BP-SeNPs prepared using aqueous pure water (0.25 mg/mL) solutions were measured by a combined dynamic light scattering (DLS) and particle electrophoresis apparatus (Zetasizer Nano ZS, Malvern Panalytical, Malvern, UK).

### 2.5. Structure Characterization of BP-SeNPs

#### 2.5.1. Spectroscopic Analysis

The sample solutions (0.1 mg/mL, 2 mL) were poured into quartz cuvettes including BP, BP-SeNPs, SeNPs, VC, and Na_2_SeO_3_ and screened on a TU-1901 UV–vis double-beam spectrophotometer (Shimadzu Corporation, Kyoto, Japan) within the wavelength range of 200–600 nm. Scanning was performed in a high-resolution mode.

Freeze-dried samples (SeNPs and BP-SeNPs) were crushed with KBr and then examined by a FTS135 FTIR spectrometer (Bio-Rad Laboratories Co., USA) with a range of 4000–400 cm^−1^. The scanning count was set to 32 with a resolution of 4 cm^−1^.

#### 2.5.2. Microstructure and Element Composition Determination

The crystal structures of BP, SeNPs, and BP-SeNPs were evaluated by a Powder X-ray diffractometer (PANalytical B.V., Almelo, The Netherlands) with Cu-Ka radiation (40 kV, 35 mA) over a 2θ range of 5–90° at a scanning speed of 2° / min^−1^.

The purity and elemental composition of BP-SeNPs were tested by the scanning electron microscope with energy-dispersive X-ray analysis (S-3400, Hitachi, Ltd., Tokyo, Japan). The samples were sputter-coated with gold prior to imaging, with an acceleration voltage set to 5 kV.

#### 2.5.3. Morphological Observation

The morphological features of the two samples (SeNPs and BP-SeNPs) were observed under different magnifications using SEM (S-3400, Hitachi, Ltd., Tokyo, Japan) and TEM (H-7650, Hitachi, Ltd., Tokyo, Japan). For TEM analysis, the samples were prepared by dropping the diluted solutions on a copper plate covered with carbon film and then viewed at accelerating voltage of 200 kV after drying.

### 2.6. Stability Measurement of BP-SeNPs

After passing the 0.45 μm water membrane, BP-SeNPs solution (0.25 mg/mL) was stored in 4 °C for 0, 7, 14, 21, and 28 days, respectively. The storage stability was analyzed by measuring diameters of BP-SeNPs. Similarly, the stabilities of BP-SeNPs solutions were determined at different temperatures (in 4, 30, 60, and 90 °C thermostatic bath), different pH (2, 4, 6, 8, and 10) adjusted by 1.0 mol/L HCl and 1.0 mol/L NaOH, and different ion strengths (0.01, 0.05, 0.10, 0.20, and 0.40 mol/L NaCl).

### 2.7. Determination of the Effects of BP-SeNPs on Erythrocyte Hemolysis

#### 2.7.1. Hemolysis Rate Analysis Damaged by AAPH

For the preparation of a 300 mmol/L AAPH stock solution, 2.033 g of AAPH was accurately weighed into a dry beaker. Fifteen millilitres of pre-chilled PBS (pH 7.4) was added, and the mixture was sonicated to ensure complete dissolution. The solution was then transferred to a 25 mL volumetric flask and brought to volume with PBS.

The 2% rabbit erythrocytes suspension (1.0 mL) was diluted to 1% erythrocytes suspension with PBS (10 mmol/L, pH 7.4), then mixed with 2.0 mL AAPH (6.25–200 mmol/L), and incubated at 37 °C for reaction. Every 0.5 h, 200 μL of the mixed solution (diluted 20 times with PBS) was collected and centrifuged at 715× *g* for 10 min to separate erythrocytes from serum. The supernatant was collected and the absorbance (*A*_1_) was recorded at 540 nm. Similarly, 200 μL aliquot of the same reaction mixture was collected each time and diluted 20 times with deionized water (instead of PBS) to induce complete hemolysis. After incubation to ensure full lysis, the solution was centrifuged under the same conditions. The absorbance (*A*_2_) of the resulting supernatant was then measured at 540 nm using deionized water as the blank. The hemolysis rate was calculated using the following equation:

(1) $$\mathrm{Hemolysis}\;\mathrm{rate}\;(\%)\;=\;\frac{A_{1}}{A_{2}}\;\times \;100$$

#### 2.7.2. Inhibitory Activity of BP-SeNPs Against Erythrocytes Hemolysis

The inhibitory activity of BP-SeNPs on AAPH-induced hemolysis was determined as described in our previous study. Briefly, 1.0 mL of 2% rabbit erythrocytes suspension was mixed with 1.0 mL sample (SeNPs, BP, BP-SeNPs, and VC) with different concentrations (0.125, 0.25, 0.5, 1.0, and 2.0 mg/mL, respectively). After pre-incubation at 37 °C for 20 min, oxidative stress was induced by adding 2.0 mL of 200 mmol/L AAPH solution, followed by incubation at 37 °C for 2 h. Subsequently, 200 µL of the reaction mixture was withdrawn, diluted 20-fold with PBS, and centrifuged at 715× *g* for 10 min. The absorption values of *A*_1_ and *A*_2_ were determined according to the procedure in Section 2.7.1. Then, the inhibition rate of samples against hemolysis was calculated [33].

#### 2.7.3. Mechanism of BP-SeNPs Against Erythrocytes Hemolysis

The contents of MDA and SA, the activities of key antioxidant enzymes (GSH-Px, CAT, and SOD) and ATPases (Na^+^/K^+^-ATPase, Ca^2+^-ATPase, and Mg^2+^-ATPase), were determined using MDA Assay Kit (cat. no. ab118970; Abcam, Cambridge, UK), SA Assay Kit (cat. no. ab83375, abcam), GSH Assay Kit (cat. no. ab239727; Abcam, Cambridge, UK), CAT Assay Kit (cat. no. ab83464; Abcam, Cambridge, UK), SOD Assay Kit (cat. no. ab65354; Abcam, Cambridge, UK), Na^+^/K^+^-ATPase assay kit (cat. no. BC0065), Ca^2+^-ATPase assay kit (cat. no. BC0960), and Mg^2+^-ATPase assay kit (cat. no. BC0960), respectively, in accordance with the manufacturers’ protocol [34,35]. Erythrocytes of different treatment groups were incubated according to the procedure described in Section 2.7.2. The experiment was divided into a blank group (erythrocytes incubated in PBS), a negative control group (erythrocytes treated with AAPH alone), experimental groups (erythrocytes pre-incubated with 0.125–2.0 mg/mL SeNPs, BP, and BP-SeNPs, respectively, prior to AAPH treatment), and positive control groups (erythrocytes pre-incubated with 0.125–2.0 mg/mL VC prior to AAPH treatment).

The MDA content was determined by the thiobarbituric acid (TBA) reaction method, wherein MDA reacts with TBA under acidic and high temperature conditions to form a pink chromogen, whose absorbance was measured at 532 nm. GSH-Px activity was measured by quantifying the rate of GSH consumption during the enzymatic reduction of H_2_O_2_. CAT activity was assessed by monitoring H_2_O_2_ decomposition at 240 nm. SOD activity was evaluated based on its inhibition of nitroblue tetrazolium reduction by superoxide anion generated from the xanthine/xanthine oxidase system, with absorbance read at 560 nm.

The activities of ATPases were quantified by measuring the amount of inorganic phosphate (Pi) liberated from ATP hydrolysis, using a molybdate-based colorimetric assay at 660 nm. SA content was determined based on its reaction with 5-methylresorcinol in the presence of an oxidant to form a purple-red complex with maximum absorption at 560 nm; absorbance at the wavelength was used for calculation. All concentrations involved in this study were the mass concentration of the sample (BP, SeNPs, or BP-SeNPs mass).

### 2.8. Statistical Analysis and Figure Plotting

The above experiments were replicated three times (technical or biological replicates); the results were shown as mean value ± standard deviation (SD). Statistical analyses were performed using IBM SPSS Statistics software (version 23.0; IBM Corp., Armonk, NY, USA). Differences between groups were assessed by one-way analysis of variance (ANOVA), with *p* < 0.05 considered statistically significant. All figures were generated using Origin software (2017 version, OriginLab Corporation, Northampton, MA, USA).

## 3. Results and Discussion

### 3.1. Characterization of BP

#### 3.1.1. Physicochemical Properties of BP

The crude polysaccharides from blueberry pomace with a purity of 25.62 ± 1.14% were obtained using ultrasonic-assisted ammonium aqueous two-phase method. After isolating BP-SeNPs, which showed better dispersion and smaller size compared to SeNPs, a polysaccharide named BP was obtained with a purity of 72.18 ± 1.97% using D101 macroporous resin. Qualitative analysis indicated that BP contained 28.00 ± 0.12% uronic acid, identifying it as an acidic polysaccharide. Additionally, BP contained only trace amounts of protein (0.89 ± 0.02%) and polyphenol (0.10 ± 0.01%).

**Figure 2:**
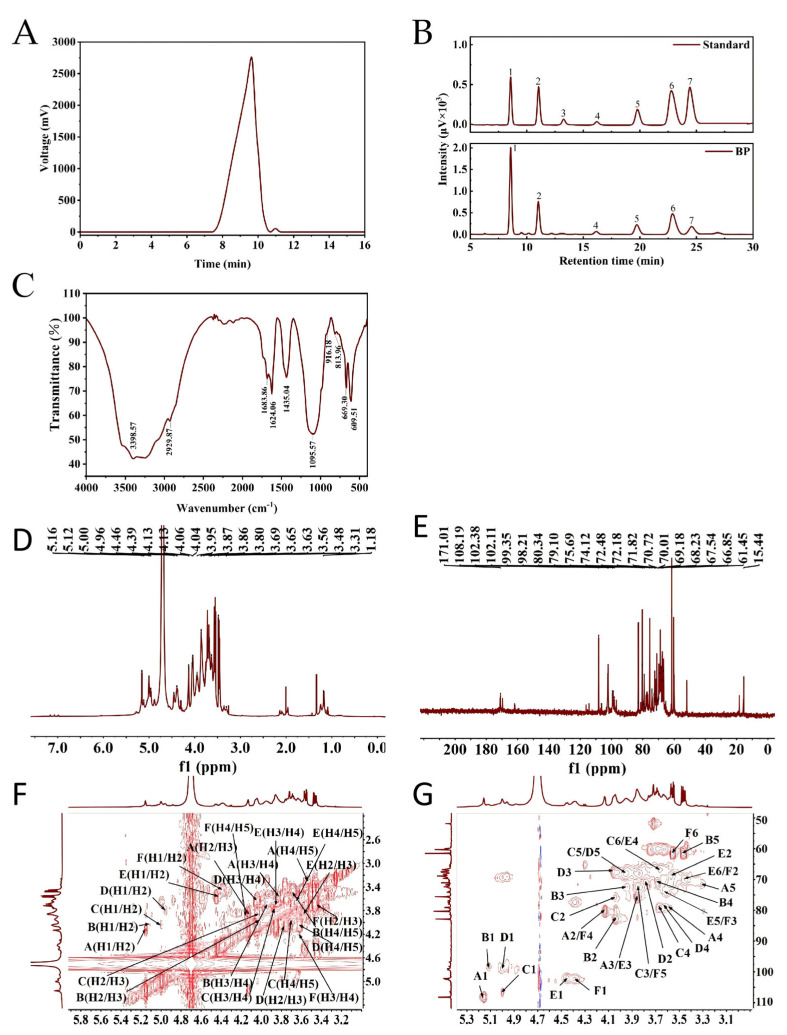
Characterization of BP. (**A**) Elution curve of BP on gel permeation chromatography; (**B**) HPLC elution profile of mixed monosaccharide standards and BP (1: mannose; 2: rhamnose; 3: glucuronic acid; 4: galacturonic acid; 5: glucose; 6: galactose; and 7: arabinose); (**C**) FT-IR spectrum; (**D**) ^1^H NMR spectrum; (**E**) ^13^C NMR spectrum; (**F**) ^1^H-^1^H COSY NMR spectrum; and (**G**) ^1^H-^13^C HSQC NMR spectrum.

As shown in Figure 2A, BP presented a single symmetrical peak at 9.62 min in elution diagram, which indicated that the molecular mass distribution range of BP was relatively narrow. Based on the standard curve (lg*M*_w_ = −0.400*t* + 9.586, *R*^2^ = 0.991), the *M*_w_ of BP was estimated as 5.4 × 10^5^ Da. Monosaccharide composition analysis showed that BP was composed of d-mannose, l-rhamnose, d-galacturonic acid, d-glucose, d-galactose, and l-arabinose, with the percentage mass ratios of 36.09:18.94:15.56:14.26:9.83:5.32, respectively (Figure 2B). Hu et al. [36] reported that a polysaccharide fraction (BLP-1) from blueberry leaves was extracted using ultrasound-assisted and hot water extraction. It was mainly composed of Rha, Ara, Gal, Glc, Xyl, Man, and GlcA, with molar ratios of 0.004:0.127:0.396:0.415:0.041:0.007. The difference in monosaccharide composition may be explained by the different extraction methods, because the physicochemical properties were affected by the extracting procedure [37].

#### 3.1.2. FT-IR Spectrum

The FT-IR spectrum of BP was shown in Figure 2C; a broad band at 3398.57 cm^−1^ and a weak band at 2929.87 cm^−1^ were assigned to the stretching vibration of O-H and C-H, respectively [38], while the intense absorption peaks around 1683.86 and 1624.06 cm^−1^ were attributed to C=O and COO^−^ stretching vibration, respectively [39]. The absorption peaks between 1000 and 1200 cm^−1^ were the typical characteristic of the pyranose ring vibrations [40]. Furthermore, the existence of β-glycoside and α-glycoside linkages was also demonstrated by the absorption regions at 916.18 and 813.96 cm^−1^ [31].

#### 3.1.3. NMR Analysis

Figure 2D,E showed the 1D NMR spectra of BP; the six anomeric proton signals (*δ*_H_ 5.16, 5.12, 5.00, 4.96, 4.46, and 4.39 ppm) and six anomeric carbon signals (*δ*_C_ 108.19, 106.48, 102.38, 102.11, 99.35, and 98.22 ppm) confirmed the existence of both α-linked and β-linked glycosidic bonds and six sugar residues [40]. The chemical shift in *δ*_H_ 1.17 and *δ*_C_ 15.44 ppm were attributed to the CH_3_ group of rhamnose [41], and the signal of *δ*_C_ 171.01 ppm indicated the presence of uronic acid [42].

The configuration and arrangement of sugar residues in BP were further analyzed using 2D NMR; HSQC spectrum (Figure 2F) showed the presence of six cross-peaks including *δ*_H/C_ 5.16/108.19, 5.12/98.22, 5.00/106.48, 4.96/99.35, 4.46/102.38, and 4.39/102.11 ppm and labelled as residues A–F in order. For residue A, the chemical shifts in H2–H5 were assigned to *δ*_H_ 4.13, 3.85, 3.56, and 3.31 ppm by correlating the related signals detected at *δ*_H/H_ 5.16/4.13, 4.13/3.85, 3.85/3.56, and 3.56/3.31 ppm in the ^1^H-^1^H COSY spectrum (Figure 2G). At the same time, coupling peaks of H2/C5–H5/C5 at *δ*_H/C_ 5.16/108.19, 4.13/80.34, 3.85/77.57, 3.56/79.38, and 3.31/71.82 ppm were found in HSQC spectrum. So, the residue A was identified as →5)-α-l-Ara*f*-(1→ [40,43]. Using the same method, the chemical shifts in other C and H atoms in residues B–F (→2)-*α*-l-Rha*p*-(1→, →4)-*β*-d-Man*p*-(1→, →4)-*α*-d-Gal*p*A-(1→, →3, 6)-*β*-d-Gal*p*-(1→, and →4)-*β*-d-Glc*p*-(1→) were confirmed and recorded in Table 1.

### 3.2. Structure Characteristics of BP-SeNPs

#### 3.2.1. Particle Size and Zeta Potential

**Figure 3:**
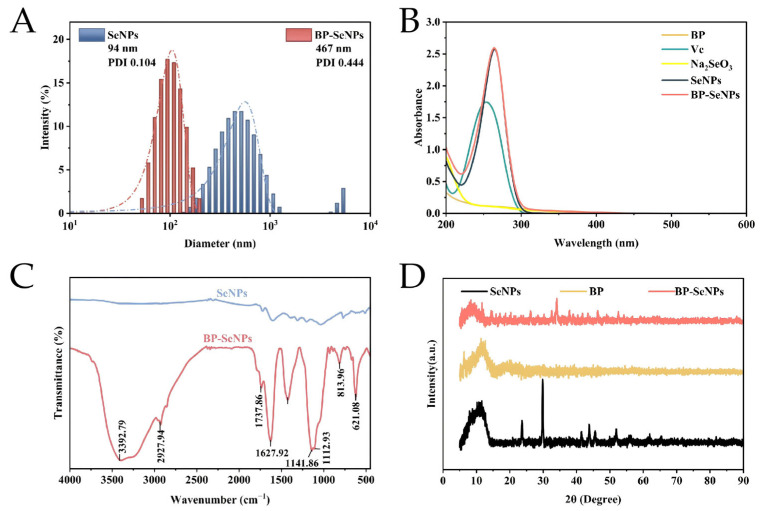
Structural characterization of SeNPs and BP-SeNPs. (**A**) Particle size distribution; (**B**) UV–vis absorption spectra; (**C**) FT-IR spectra; and (**D**) XRD spectra.

As shown in Figure 3A, the average particle size of SeNPs was 486.95 ± 18.55 nm. When BP was used as a template, the particle size was decreased to 94.33 ± 0.60 nm, indicating that the BP in the solution acted as a stabilizer and prevented the aggregation of the selenium nanoparticles. The similar results were obtained as the SeNPs were prepared with *Chaenomeles speciosa* polysaccharide [48], *Polyporus umbellatus* polysaccharide [13], and *Usnea longissima* polysaccharide [49]. In addition, the PDI of BP-SeNPs was 0.104, which was markedly lower than that of SeNPs (0.444), indicating a narrower particle-size distribution and improved dispersion stability. The zeta potentials of SeNPs and BP-SeNPs were −2.65 ± 0.24 and −16.38 ± 0.77 mV, further evidencing the better stability of BP-SeNPs.

#### 3.2.2. UV–Vis Spectra

The UV-vis spectra of VC, Na_2_SeO_3_, BP, SeNPs, and BP-SeNPs were shown in Figure 3B; VC showed an absorption peak at 253.5 nm, while Na_2_SeO_3_ and BP had no characteristic bands. The same absorption peaks appeared separately at 264.0 nm in SeNPs and BP-SeNPs systems, proving the formation of selenium nanoparticles, which was consistent with the results in previously reported articles [8,50].

#### 3.2.3. FT-IR Spectra

FT-IR was applied to clarify the possible combination between selenium and BP, as shown in Figure 3C. SeNPs had no absorption peaks in the functional group region (4000–1500 cm^−1^). BP-SeNPs had the similar absorption peaks to that of BP, but the characteristic peak of the O-H stretching shifted from 3399 cm^−1^ (BP, Figure 2C) to 3393 cm^−1^ (BP-SeNPs), and the peak intensity decreased; this red shift might be attributed to the interaction between -OH and SeNPs [51]. The surface of selenium was strongly adsorbed and passivated by BP molecules, resulting in a steady BP-SeNPs system [52]. Mikhailova [53] also reported the same conclusion that the Ganoderma lucidum polysaccharide bonded with SeNP.

#### 3.2.4. XRD Analysis

XRD was applied to study the crystal pattern of BP-SeNPs. As shown in Figure 3D, BP was an amorphous structure based on the only broad diffraction peak at 2θ = 11°. SeNPs exhibited intensive and sharp diffraction peaks at around 23.5, 29.8, 41.4, 43.7, and 45.4, and 51.7° corresponded to the (100), (101), (110), (102), (111), and (201) reflections of pure hexagonal Se crystals (ICDD PDF# 06-0362). However, the two typical peaks at 23.5 ° and 29.8 ° were weakened significantly after the introduction of BP into the redox system, which suggested that the SeNPs were adorned by BP, causing the emergence of amorphous SeNPs. Concórdio-Reis et al. [54] synthetized SeNPs using the novel exopolysaccharide (EPS) produced by the marine bacterium *Alteromonas macleodii* Mo 169 and reported the same change pattern.

#### 3.2.5. EDX Analysis

**Figure 4:**
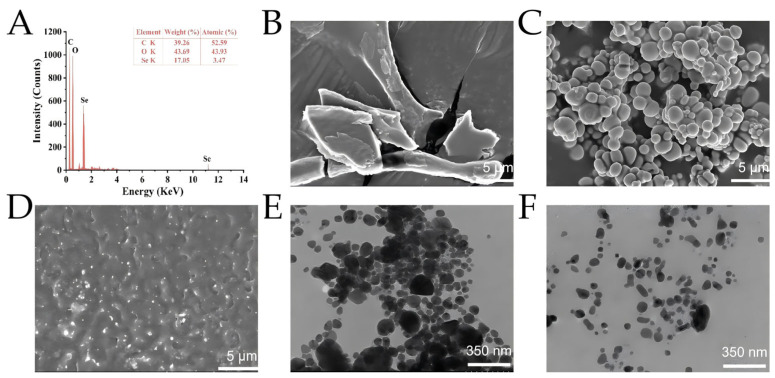
EDX, SEM, and TEM analysis of BP-SeNPs. EDX spectrum of BP-SeNPs (**A**); SEM images of BP (**B**), SeNPs (**C**), and BP-SeNPs (**D**); and TEM images of SeNPs (**E**) and BP-SeNPs (**F**).

As shown in Figure 4A, EDX was applied to determine the elemental composition of BP-SeNPs; there were three signals observed, which confirmed BP-SeNPs were mainly composed of C, O, and Se elements in the atomic proportion of 52.59%, 43.93%, and 3.47%. Selenium absorption peaks were presented at 1.37 keV (SeL peak) and 11.22 keV (SeK peak) [55]. C and O elements were attributed to the presence of alkyl chains in the BP molecules. There were no obvious peaks for other elements or impurities.

#### 3.2.6. SEM and TEM Analysis

The micromorphology and microstructure of BP-SeNPs were described by SEM and TEM. The SEM images confirmed that BP presented a flake-like structure with a rough surface (Figure 4B); SeNPs existed in the state of aggregation with the sphere-like morphology (Figure 4C), and BP-SeNPs had more dispersion and a smaller size compared to SeNPs (Figure 4D). TEM images also confirmed that BP had a significant effect on the formation and growth of selenium nanoparticles. SeNPs had a number of spherical particles gathered into a large agglomeration (Figure 4E), while BP-SeNPs presented a monodisperse uniform spherical structure surrounded by polysaccharides (Figure 4F). Meanwhile, the average particle sizes of SeNPs and BP-SeNPs by TEM were 459 ± 15.32 and 122 ± 3.11 nm, which were similar to the results obtained by DLS.

### 3.3. Stability of BP-SeNPs

**Figure 5:**
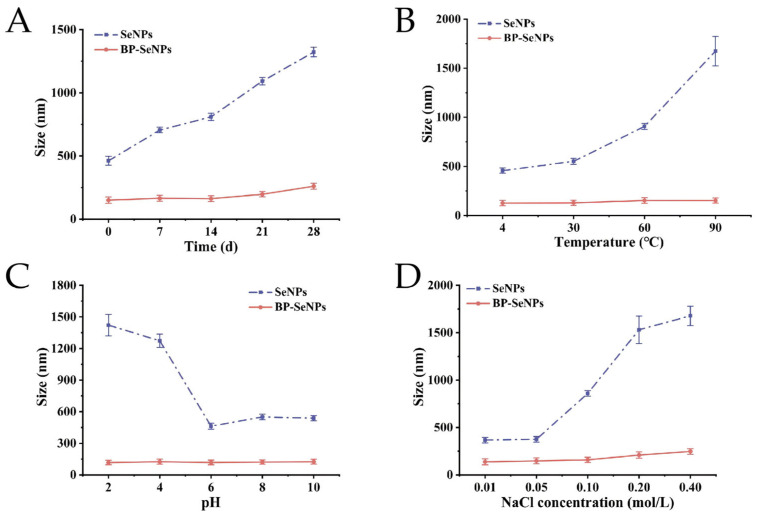
The influence of storage time (**A**), temperature (**B**), pH (**C**), and ionic strength (**D**) on the particle sizes of SeNPs and BP-SeNPs.

As shown in Figure 5A, during 28 days of storage at room temperature, the average particle size of SeNPs increased markedly from 461 nm to 1323 nm, indicating severe aggregation. In contrast, BP-SeNPs only showed a modest size increase from 138 to 247 nm, demonstrating good long-term storage stability. Similarly, Yu et al. [56] showed that SeNPs stabilized by longan polysaccharides exhibited minimal aggregation over prolonged storage (14 days), with average particle sizes maintained below 150 nm. As the temperature raised from 4 °C to 90 °C (Figure 5B), the particle size of SeNPs was significantly influenced, increasing markedly from 457 nm to 1674 nm. The increase in temperature intensifies the motion of nanoparticles, leading to enhanced collisions among SeNPs and consequently promoting particle aggregation, which was consistent with the experimental observations reported by Zhang et al. [57]. BP-SeNPs also maintained stable particle sizes (100–150 nm) across a broad pH range of 2 to 10 (Figure 5C) and within ionic strength range (Figure 5D) of 0.01–0.40 mol/L. High ionic strength can shield the surface charges of nanoparticles and can reduce electrostatic repulsion, promoting aggregation [58]. BP-SeNPs resisted this effect; the reason might be that the surface of selenium was strongly adsorbed and passivated by BP molecules [59]. Polysaccharide chains provided steric hindrance, physically preventing close contact between particles. Moreover, hydroxyl and carboxyl residues contributed to electrostatic repulsion, further enhancing dispersion stability [60].

### 3.4. Inhibition Effects of BP-SeNPs on Erythrocyte Hemolysis

#### 3.4.1. Model Building of Erythrocyte Hemolysis Damaged by AAPH

**Figure 6:**
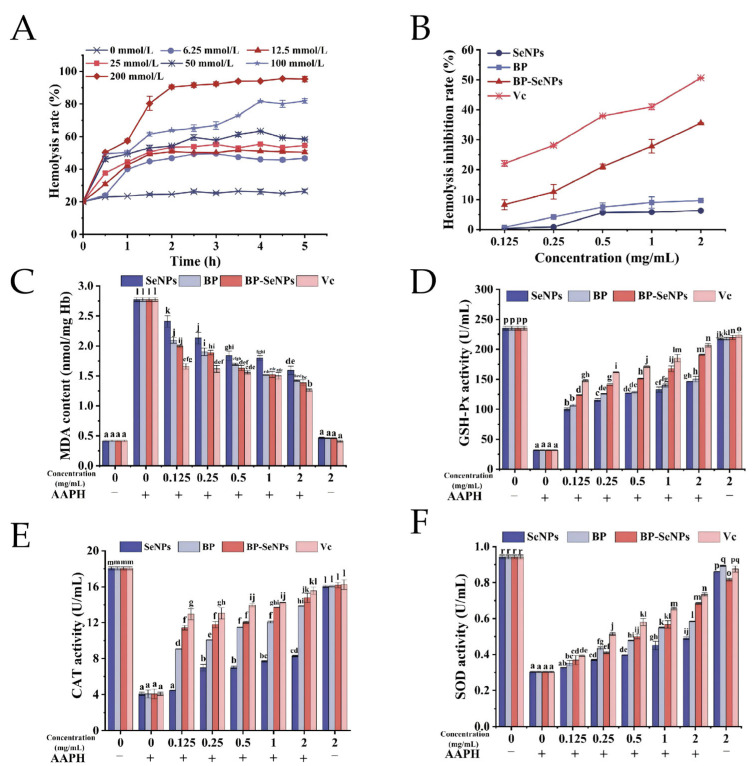
Protection effects of BP-SeNPs on erythrocyte hemolysis. (**A**) Oxidative hemolysis of erythrocytes induced by AAPH; (**B**) hemolysis inhibitory action of BP-SeNPs; effects of BP-SeNPs on MDA content (**C**), GSH-Px activity (**D**), CAT activity (**E**), and SOD activity (**F**) in AAPH-induced erythrocytes. Different letters above the bars indicate significant differences (*p* < 0.05).

The time profile for hemolysis induced using AAPH at different concentrations is shown in Figure 6A. The hemolysis rate of erythrocytes was 20.15 ± 0.76% without AAPH. After addition of AAPH, the hemolysis rate increased in a dependent manner with prolonged incubation time. At a concentration of 200 mmol/L, the hemolysis rate was 90.41 ± 1.18% within the first 2 h. Therefore, a model of oxidative damage was constructed using 200 mmol/L AAPH with an incubation time of 2 h.

#### 3.4.2. Protective Effect of BP-SeNPs on Erythrocytes Hemolysis

It was reported that nano-metal can form a protective layer by combining with cell membrane at an appropriate concentration, reducing the damage caused by external factors, and then inhibiting cell lysis [61]. The inhibition rates of the samples (SeNPs, BP, BP-SeNPs, and VC) on erythrocyte hemolysis at different concentrations are shown in Figure 6B. The results showed VC and BP-SeNPs had great protection effects on erythrocytes hemolysis; the hemolysis inhibition rate gradually increased in a dose-dependent manner (*p* < 0.05). At a concentration of 2.0 mg/mL, the inhibitory rate of BP-SeNPs was 35.53 ± 0.15%. The inhibition rate of BP-SeNPs (35.53 ± 0.15%) was lower than that of VC (50.64 ± 0.11%), but much higher than those of BP (9.70 ± 0.05%) and SeNPs (6.29 ± 0.07%) under the same conditions.

### 3.5. The Mechanism of BP-SeNPs Reducing Hemolysis Rate

#### 3.5.1. Influence of BP-SeNPs on MDA Content and Antioxidant Enzyme Activities

MDA is one of the important products of lipid peroxidation induced by free radicals attack on the cell membrane. Cross-linking of MDA with proteins and nucleic acids ultimately causes cytotoxic effects. Thus, cellular MDA level is considered an indirect indicator of oxidative damage [62]. As shown in Figure 6C, the MDA content of erythrocytes in blank control (without AAPH and the samples) was 0.42 ± 0.01 nmol/mg Hb, and no obvious differences were found after treatment only with the samples (BP, SeNPs, BP-SeNPs, and VC) at 2.0 mg/mL, indicating that the samples had no effects on MDA formation. When the erythrocytes were processed by AAPH, the level of MDA was as high as 2.77 ± 0.03 nmol/mg Hb. However, the pretreatment with the samples significantly alleviated MDA levels in AAPH-induced cells. The inhibition effects of the different samples on MDA formation were VC > BP-SeNPs > BP > SeNPs. When the concentration of the samples was 2.0 mg/mL, the MDA contents were reduced to 1.26 ± 0.02, 1.38 ± 0.05, 1.42 ± 0.01, and 1.59 ± 0.09 nmol/mg Hb, respectively.

Moreover, the pretreatment of BP-SeNPs had significant protective effects on the antioxidant defence system, evidenced by significant changes in SOD, CAT, and GSH-Px enzyme activities (Figure 6D–F). The GSH-Px, CAT, and SOD activities of the unprocessed erythrocytes were 235.46 ± 3.13, 18.05 ± 0.16, and 0.94 ± 0.01 U/mL, respectively. When erythrocytes were treated with AAPH only, GSH-Px activity decreased to 31.83 ± 0.64 U/mL, CAT activity decreased to 4.07 ± 0.18 U/mL, and SOD activity decreased to 0.30 ± 0.003 U/mL, suggesting that the antioxidant system of erythrocytes was significantly destroyed. When the concentration of BP-SeNPs was 2.0 mg/mL, the activities of SOD, CAT, and GSH-Px enzyme increased to 172.98 ± 3.58, 13.86 ± 0.03, and 0.58 ± 0.003 U/mL, respectively. In addition, the enzymatic activities in the erythrocytes treated only with samples (BP, SeNPs, BP-SeNPs, and VC) were similar to that of the blank control, showing that the samples did not affect enzymes related to oxidative stress. At the same time, the contents of three enzymes showed a dose-dependent relationship with BP, SeNPs, BP-SeNPs, and VC concentrations (*p* < 0.05). Furthermore, the order of restoration of samples on GSH-Px, CAT, and SOD activities were as follows: VC > BP-SeNPs > BP > SeNPs.

#### 3.5.2. Influence of BP-SeNPs on Erythrocyte Membranes

**Figure 7:**
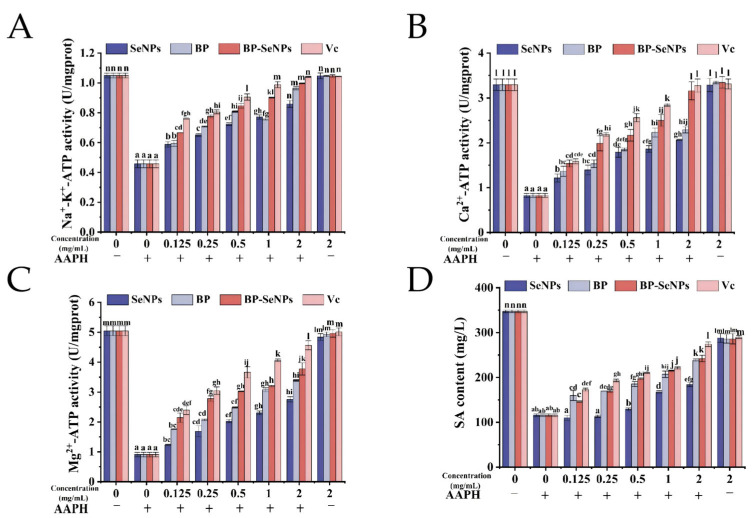
Effects of BP-SeNPs on Na^+^/K^+^-ATPase (**A**), Ca^2+^-ATPase (**B**), Mg^2+^-ATPase (**C**) activities, and SA content (**D**). Different letters above the bars indicate significant differences (*p* < 0.05).

ATPases, mainly including Na^+^/K^+^-ATPase, Mg^2+^-ATPase, and Ca^2+^-ATPase, were functional proteins that regulate the ion homeostasis between the inside and outside of erythrocyte membrane. When an organism undergoes oxidative stress, the activity of ATPases decreases, which may affect the function of cell membranes [63]. As shown in Figure 7, the activities of Na^+^/K^+^-ATPase, Ca^2+^-ATPase, and Mg^2+^-ATPase in normal erythrocytes (the blank group) were 1.05 ± 0.02, 3.29 ± 0.13, and 5.05 ± 0.16 U/mg prot, respectively. When the erythrocytes were incubated with 2.0 mg/mL SeNPs, BP, BP-SeNPs, or VC alone, ATPase activity remained at a background level and basically equalled to the blank group. Compared with the blank group, the activities of Na^+^/K^+^-ATPase, Ca^2+^-ATPase, and Mg^2+^-ATPase decreased to 0.46 ± 0.03, 0.83 ± 0.04, and 0.91 ± 0.08 U/mg prot after AAPH-induced oxidative damage. The activity of all three ATPases were recovered to varying degrees and showed a concentration dependence after the addition of VC, BP-SeNPs, BP, and SeNPs. With the injection of 2.0 mg/mL of BP-SeNPs, the activities of Na^+^/K^+^-ATPase, Ca^2+^-ATPase, and Mg^2+^-ATPase were 1.00 ± 0.01, 3.16 ± 0.20, and 3.78 ± 0.21 U/mgprot, respectively, indicating that BP-SeNPs can effectively alleviate damage. Under the same conditions, BP-SeNPs were slightly more effective than SeNPs in restoring the activity of these ATPases.

SA, on the cell surface as a glycoprotein or glycolipid, played a crucial role in regulating intercellular recognition, immune responses, and synaptic transmission. In addition, SA was a major source of negative charge on the cell membrane surface. As can be seen from Figure 7D, the SA content in normal rabbit red blood cells was 347.46 ± 3.04 mg/L; when erythrocytes mixed with AAPH, the SA content dropped to 115.76 ± 2.98 mg/L. However, when the samples (BP-SeNPs, VC, BP, and SeNPs) were added and incubated with erythrocytes for 2.0 h, the SA content gradually increased in a dose-dependent manner (*p* < 0.05). At a sample concentration of 2.0 mg/mL, the SA contents were 242.28 ± 7.17, 273.97 ± 5.09, 238.58 ± 2.77, and 184.11 ± 4.27 mg/L, respectively, suggesting that the samples had a protective effect on erythrocytes and obviously prevented the reduction in SA content.

Based on the result of Section 3.5, BP-SeNPs protected enzyme activities and increased ATPase and SA contents against AAPH-induced reduction. Under the same conditions, BP-SeNPs exhibited better effect than SeNPs; this result was consistent with other research findings. For example, the antioxidant activity of SeNPs modified by chestnut polysaccharide was improved [64]. SeNPs prepared using the polysaccharides from *Ribes nigrum* L. (RP) showed significantly better antiglycation and *α*-glucosidase inhibitory activity than pure SeNPs [65]. The SeNPs dispersed by polysaccharides presented enhanced bioactivity, which may be attributed to the following factors: as a stabilizer, polysaccharides can effectively protect SeNPs particles from aggregation through the interaction of Se–O bonds, thereby maintaining their high activity. This effect can be confirmed by the smaller particle size and higher absolute potential of SeNPs after polysaccharide modification [65]. At the same time, the particle size was reduced, resulting in a significant increase in surface area, enabling more efficient scavenging of free radicals [49]. Polysaccharides inherently possess excellent biocompatibility and degradability, facilitating the absorption and utilization of SeNPs. In the present study, BP-SeNPs increased ATPase and SA contents greatly, which may be attributed to the interaction between polysaccharides and lipid molecules in the cell membrane; this improved membrane stability and reduced the opportunity for AAPH molecules or free radicals to interact with the cell membrane.

## 4. Conclusions

In the present study, a polysaccharide (BP) was successfully extracted from blueberry pomace and utilized as a dispersant to synthesize BP-SeNPs. Compared to bare SeNPs, the BP-SeNPs exhibited a significantly smaller particle size, improved monodispersity, and substantially enhanced stability, validating our initial hypothesis that plant polysaccharides can effectively prevent nanoparticle aggregation. Furthermore, the in vitro erythrocyte hemolysis assay demonstrated that BP-SeNPs possess superior protective effects compared to SeNPs alone. The underlying mechanism was elucidated: BP-SeNPs effectively mitigated oxidative damage by enhancing intracellular antioxidant enzyme activities (SOD, CAT, and GSH-Px), reducing lipid peroxidation (MDA level), and preserving membrane integrity through the maintenance of ATPase activities and sialic acid content. Our work not only provides a novel and efficient method for preparing stable SeNPs using agricultural by-product polysaccharides but also expands the potential application of BP-SeNPs as promising selenium nutritional supplements in the fields of functional foods and nutraceuticals. Future studies should focus on exploring their in vivo bioavailability, long-term safety profile, and more detailed molecular mechanisms of action in relevant disease models.

## Figures and Tables

**Table 1:** ^1^H and ^13^C NMR signal assignments of the residues in BP.

Sugar Residues	Chemical Shifts ^1^H/^13^C (ppm)						
	H1/C1	H2/C2	H3/C3	H4/C4	H5/C5	H6/C6	References
**A** →**5)-α-l-Ara*f*-(1**→	5.16/108.19	4.13/80.34	3.85/77.57	3.56/79.38	3.31/71.82	–/–	[40,43]
**B** →**2)-**α**-l-Rha*p*-(1**→	5.12/98.22	4.04/82.97	3.95/72.48	3.69/71.24	3.48/61.45	1.17/15.44	[44]
**C** →**4)-**β**-d-Man*p*-(1**→	5.00/106.48	4.04/75.69	3.87/72.18	3.69/79.10	3.95/68.23	3.65/66.85	[45,46]
**D** **4)-α-d-Gal*p*A-(1**→	4.96/99.35	3.80/70.72	4.06/67.54	3.64/78.40	3.95/68.23	–/171.01	[44]
**E** →**3,6)-**β**-d-Gal*p*-(1**→	4.46/102.38	3.56/69.18	3.85/77.57	3.65/66.85	3.64/74.12	3.48/70.01	[40]
**F** →**4)-**β**-d-Glc*p*-(1**→	4.39/102.11	3.48/70.01	3.64/74.12	4.13/80.34	3.87/72.18	3.56/61.45	[47]

## Data Availability

The original contributions presented in the study are included in the article. Further inquiries can be directed to the corresponding authors.
